# Circulatory Indicators of Lipid Peroxidation, the Driver of Ferroptosis, Reflect Differences between Relapsing–Remitting and Progressive Multiple Sclerosis

**DOI:** 10.3390/ijms252011024

**Published:** 2024-10-14

**Authors:** Ljiljana Stojkovic, Ana Djordjevic, Milan Stefanovic, Aleksandra Stankovic, Evica Dincic, Tamara Djuric, Maja Zivkovic

**Affiliations:** 1 Laboratory for Radiobiology and Molecular Genetics, VINČA Institute of Nuclear Sciences—National Institute of the Republic of Serbia, University of Belgrade, P.O. Box 522, 11000 Belgrade, Serbia; ana.djordjevic@vin.bg.ac.rs (A.D.); alexas@vin.bg.ac.rs (A.S.); tamariska@vin.bg.ac.rs (T.D.); 2 Clinic for Neurology, Military Medical Academy, 11000 Belgrade, Serbia; evica.vma@gmail.com; 3 Medical Faculty of the Military Medical Academy, University of Defence, 11000 Belgrade, Serbia

**Keywords:** multiple sclerosis, progression, ferroptosis, lipid peroxidation, glutathione antioxidant defense, iron metabolism

## Abstract

Ferroptosis, a lipid peroxidation- and iron-mediated type of regulated cell death, relates to both neuroinflammation, which is common in relapsing-remitting multiple sclerosis (RRMS), and neurodegeneration, which is prevalent in progressive (P)MS. Currently, findings related to the molecular markers proposed in this paper in patients are scarce. We analyzed circulatory molecular indicators of the main ferroptosis-related processes, comprising lipid peroxidation (malondialdehyde (MDA), 4-hydroxynonenal (4-HNE), and hexanoyl–lysine adduct (HEL)), glutathione-related antioxidant defense (total glutathione (reduced (GSH) and oxidized (GSSG)) and glutathione peroxidase 4 (GPX4)), and iron metabolism (iron, transferrin and ferritin) to estimate their contributions to the clinical manifestation of MS and differences between RRMS and PMS disease course. In 153 patients with RRMS and 69 with PMS, plasma/serum lipid peroxidation indicators and glutathione were quantified using ELISA and colorimetric reactions, respectively. Iron serum concentrations were determined using spectrophotometry, and transferrin and ferritin were determined using immunoturbidimetry. Compared to those with RRMS, patients with PMS had decreased 4-HNE (median, 1368.42 vs. 1580.17 pg/mL; *p* = 0.03). Interactive effects of MS course (RRMS/PMS) and disease-modifying therapy status on MDA (*p* = 0.009) and HEL (*p* = 0.02) levels were detected. In addition, the interaction of disease course and self-reported fatigue revealed significant impacts on 4-HNE levels (*p* = 0.01) and the GSH/GSSG ratio (*p* = 0.04). The results also show an association of MS course (*p* = 0.03) and EDSS (*p* = 0.04) with GSH levels. No significant changes were observed in the serum concentrations of iron metabolism indicators between the two patient groups (*p* > 0.05). We suggest circulatory 4-HNE as an important parameter related to differences between RRMS and PMS. Significant interactions of MS course and other clinically relevant parameters with changes in redox processes associated with ferroptosis support the further investigation of MS with a larger sample while taking into account both circulatory and central nervous system estimation.

## 1. Introduction

Ferroptosis has recently been highlighted as an intriguing component in the pathogenesis of neurological disorders [1]. It is a type of regulated cell death triggered by the iron-mediated accumulation of lipid reactive oxygen species (ROS) that is dependent on the inactivation of the cellular antioxidant defense system, cystine–glutamate antiporter Xc−glutathione (GSH)-glutathione peroxidase 4 (GPX4) [2,3]. Typical features of ferroptosis, such as the shrinkage of mitochondria and accumulation of lipid ROS, have been found in CNS tissues early in the course of experimental autoimmune encephalomyelitis (EAE), suggesting that ferroptosis is an early event in the pathogenesis of multiple sclerosis (MS) that could promote T-cell activation-induced inflammation and neurodegeneration as key pathogenic mechanisms of this disease [4]. Initial experiments involving the administration of agents acting as iron chelators or scavengers of lipid peroxides showed favorable reductions in inflammation, microgliosis, demyelination, and neuronal damage in models through ferroptosis inhibition [5,6,7,8], favoring their possible use in the treatment of MS.

There is evidence that individual molecular indicators of the main ferroptosis processes, encompassing lipid peroxidation, GSH-related antioxidant defense, and iron metabolism, could be associated with MS. In the human body, the highest levels of polyunsaturated fatty acids (PUFAs), which are prone to lipid peroxidation, are found in the brain [9]. Enzymatic or nonenzymatic decomposition of PUFAs leads to the generation of secondary lipid peroxidation products that can serve as molecular indicators of the lipid peroxidation process [10]. The levels of the commonly investigated secondary lipid peroxidation product malondialdehyde (MDA) were increased in the blood, and cerebrospinal fluid (CSF) of patients with MS compared to healthy individuals [11,12,13] and were positively correlated with disability in patients, while they were inversely correlated with the administration of immunomodulatory therapy [12]. The involvement of lipid peroxidation in the pathogenesis of MS was suggested by a significant association demonstrated between magnetic resonance imaging (MRI) parameters and MDA in the CSF of newly diagnosed patients with MS [14]. Mice with EAE had elevated CNS levels of another molecular indicator of lipid peroxidation, 4-hydroxynonenal (4-HNE), along with MDA, whereas detoxification of these reactive aldehydes ameliorated the disease [15]. In a model of progressive MS (PMS)-EAE, 4-HNE was increased, together with neuroinflammatory markers in the brain [16]. Complementary to reactive aldehydes as late-stage products of lipid peroxidation, this process can be monitored through the levels of circulating hexanoyl–lysine adduct (HEL), a more recently discovered early-stage lipid peroxidation indicator [17]. The levels of HEL have been correlated with lipid peroxidation-mediated inflammatory processes in autoimmune and allergic diseases [18,19].

A close link has been established between lipid peroxidation and GSH depletion in neurological diseases [20]. This is because GSH (reduced glutathione) is a cofactor for GPX4, an antioxidant enzyme that specifically quenches lipid hydroperoxides and whose reduced activity due to a lack of GSH disturbs the intracellular redox balance, leading to ferroptosis [21]. The induction of EAE triggers a significant decrease in the GSH-to-GSSG (oxidized glutathione) ratio, along with an increase in the clinical score and lipid peroxide levels in the brain, spinal cord, and blood [22]. Neuroimaging has shown that, in comparison with healthy controls, patients with relapsing–remitting (RR) MS exhibited a decline in brain GSH levels, which represents a significant predictor of worse memory and higher levels of fatigue [23,24].

An early study indicated that blood–brain barrier (BBB) dysfunction predisposes an individual to a significant increase in the concentration of free iron in the brain, followed by lipid peroxidation [25]. Excessive iron accumulation in oligodendrocytes, followed by increased lipid ROS production, was associated with oligodendrocyte and neuronal death in models of MS, indicating ferroptosis as a pivotal mechanism of demyelination and neurodegeneration in MS [26,27,28]. In addition, the presence of iron rim lesions has been associated with more severe brain tissue destruction manifested through the accumulation of clinical disability and elevated levels of circulating neurofilament light chain or increased intrathecal immunoglobulin G synthesis [29,30]. A significant relation between serum and brain iron concentration was also noticed in MS patients [31]. Iron dysregulation, as established in neurodegenerative disorders, implies changes in the molecular components that affect cellular iron homeostasis processes, including the transport, utilization, and storage of iron [3,32]. The proteins transferrin and ferritin are key components involved in the regulation of iron transport and storage, respectively [33,34]. Macrophages and oligodendrocyte lineage cells have been found to take up transferrin-bound iron during the early-onset stage of chronic (CH)-EAE, while ferritin steadily increases in the spinal cord tissue at the peak and in progressive stages of CH-EAE compared to RR-EAE [35]. In patients with MS, the levels of transferrin and ferritin in the CNS and the periphery differ with regard to multiple factors, such as the type/number of brain lesions, the disease course and severity, and administration of disease-modifying therapy, making the obtained results less coherent [36,37,38,39,40,41,42,43].

It has been shown that ferroptosis is associated with both neuroinflammation, as a principal pathogenic mechanism in RRMS, and demyelination-mediated neurodegeneration, which is prevalent in the course of progressive MS (PMS) [4,5,44]. A substantial proportion of the studies that have been conducted on MS patients so far have demonstrated differences in ferroptosis-related molecular components between the patients and healthy subjects. Most of these studies investigated the previously described molecular components in the CNS and/or the periphery and did so separately and within relatively small subject groups. Currently, there is a lack of human studies that collectively examine the molecular indicators introduced in this study, particularly in circulation, as potential ferroptosis markers that could reflect ferroptosis-related processes occurring in the affected CNS with respect to MS.

Therefore, the aim of the present study is to analyze circulatory molecular indicators of all main processes associated with ferroptosis: lipid peroxidation (MDA, 4-HNE, and HEL), GSH-related antioxidant defense (total glutathione (GSH + GSSG), GSH, GSSG, GSH/GSSG ratio and GPX4) and iron metabolism (free iron, transferrin and ferritin), in RRMS and PMS patients, in order to evaluate their contribution to the clinical manifestation of MS and differences between RR and progressive course of disease. Disease-modifying therapies and detailed demographic and clinical data were taken into account to estimate interactions with the target molecular parameters in terms of MS course and severity.

## 2. Results

### 2.1. Anthropometric, Clinical, and Molecular Parameters in MS Patients with Regard to Disease Course

The anthropometric and clinical parameters of patients regarding the course of MS are presented in Table 1.

In comparison to RR, patients with progressive disease course (PMS) (PMS—secondary progressive (SP) and primary progressive (PP)) were significantly older and had longer disease duration (Table 1). As expected, due to progressive disease, they had significantly higher expanded disability status scale (EDSS) score, multiple sclerosis severity score (MSSS), and total number of relapses. The age at disease onset was not significantly different. Patients with PMS had a significantly higher proportion of those who self-reported sensation of fatigue and a lower proportion of those who received disease-modifying therapy compared to RR patients (Table 1).

**Figure 1:**
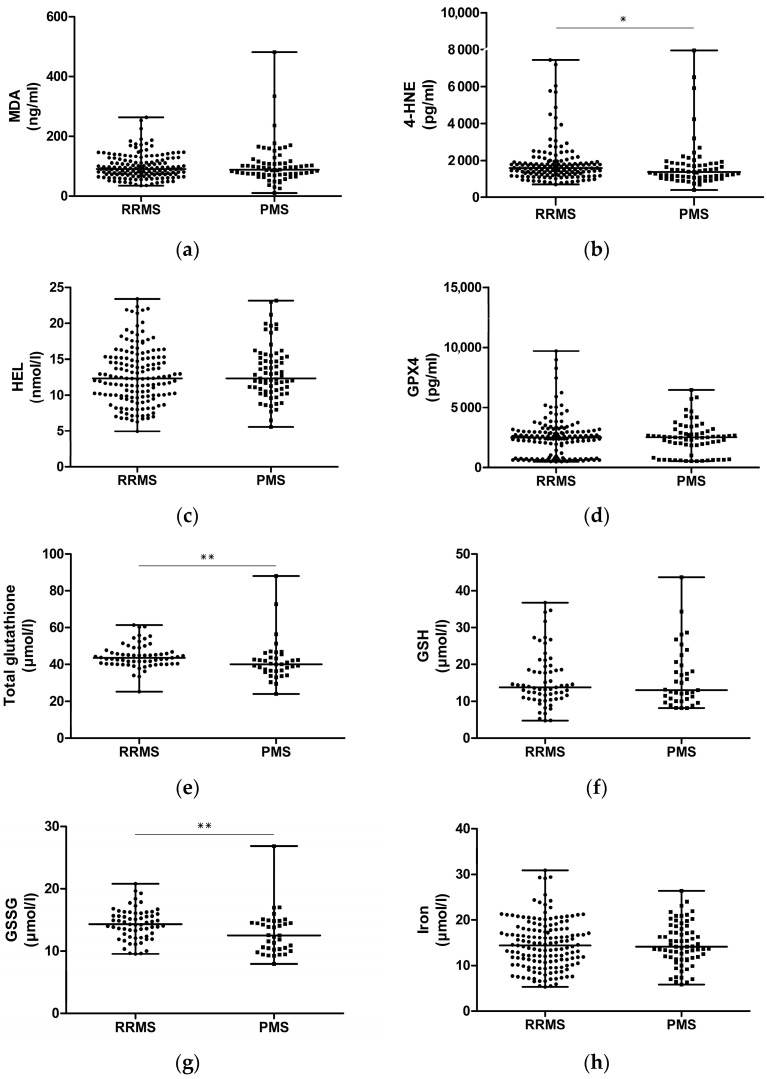

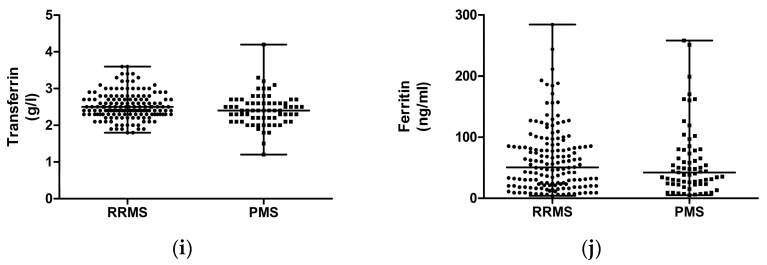
Molecular parameters in patients with RRMS and PMS: (**a**) Malondialdehyde (MDA); (**b**) 4-Hydroxynonenal (4-HNE); (**c**) Hexanoyl-lys adduct (HEL); (**d**) Glutathione peroxidase 4 (GPX4); (**e**) Total glutathione, GSH + GSSG; (**f**) Reduced glutathione (GSH); (**g**) Oxidized glutathione (GSSG); (**h**) Iron; (**i**) Transferrin; (**j**) Ferritin. RRMS—relapsing–remitting multiple sclerosis; PMS—progressive multiple sclerosis; values of parameters are presented with median and range (minimum–maximum); *p*-values (Mann–Whitney U test) < 0.05 are considered statistically significant: * *p* < 0.05, ** *p* < 0.01.

The measured molecular parameters in plasma/serum of patients with RRMS and PMS are shown in Figure 1 and Table 2. In comparison to the RRMS groups, the PMS group had significantly decreased levels of 4-HNE, *p* = 0.03), total glutathione (*p* = 0.006), and GSSG (*p* = 0.003).

### 2.2. Relationship of Anthropometric, Clinical, and Molecular Parameters in RRMS and PMS Patients

We performed univariate and multivariate analyses of the interactions between disease course and categorical parameters, including sex, smoking status, therapy status, and self-reported fatigue, regarding the levels of all the investigated molecular parameters in MS patients. Multivariate analyses showed no significant effects on any of the investigated molecular parameters (*p* > 0.05 for each).

The results for interactive effects are presented in Table 3, with the significant ones presented in Figure S1. We found a significant interactive effect of MS course and current therapy status (receiving/not receiving) on MDA levels (*p* = 0.009) (Figure S1a). Among the patients who were not receiving the therapy, those with PMS had significantly higher MDA levels (Bonferroni post hoc, *p* = 0.01).

Both RRMS and PMS patients under therapy had approximately the same levels of HEL, while among the patients who were not receiving therapy, HEL was decreased in PMS (Bonferroni post hoc, *p* = 0.04) (Figure S1b). In addition to the therapy receiving status, we took into account the major treatment types applied to the investigated groups of patients. In the RRMS group, four types of immunomodulatory treatment were used: interferon beta-1a/-1b, sphingosine-1-phosphate receptor (S1PR) modulator, anti-CD20 monoclonal antibody, and glatiramer acetate. Within this group of patients, the HEL levels were significantly lower in the patients treated with interferon beta (Bonferroni post hoc, *p* = 0.02) and glatiramer acetate (Bonferroni post hoc, *p* = 0.004) compared to those receiving the anti-CD20 antibody treatment. Regarding the two types of therapies that were used in both the RRMS and PMS groups (S1PR modulator and anti-CD20 antibody), there were no significant interactive effects of disease course and treatment type on the levels of the investigated molecular parameters (*p* = 0.56).

There was a significant interactive effect of MS course and self-reported fatigue (yes/no) on 4-HNE levels (*p* = 0.01) and the GSH/GSSG ratio (*p* = 0.04). For patients who self-reported fatigue, RRMS was associated with higher 4-HNE levels than PMS (Figure S1c) (Bonferroni post hoc, *p* = 0.009).

We analyzed the relationship between continuous clinical and anthropometric parameters and the levels of the assessed molecular parameters using disease course as a covariate (Table 4). Multivariate analyses showed no significant effect on any of the investigated molecular parameters (*p* > 0.05 for each of them). However, the univariate results indicated a significant inverse relation between EDSS and the levels of GSH (Beta = −1.43, *p* = 0.04).

We analyzed the correlations between the molecular and clinical or anthropometric parameters with respect to disease course, which are presented in Table 5. In RRMS patients (Table 5a), we found a negative correlation between GPX4 and age (R = −0.24, *p* = 0.004), while GSSG correlated positively with both age and BMI (R = 0.27, *p* = 0.03 and R = 0.29, *p* = 0.02, respectively). Furthermore, transferrin correlated negatively with disease duration and positively with MSSS (R = −0.26, *p* = 0.002 and R = 0.20, *p* = 0.01, respectively), and there was a negative correlation between ferritin and parameters of both neurological deficit (EDSS) and disease severity (MSSS) (R = −0.19, *p* = 0.02, both). In PMS patients (Table 5b), we revealed that 4-HNE correlated negatively with EDSS (R = −0.27, *p* = 0.03), and iron correlated positively with age (R = 0.30, *p* = 0.01). In both patient groups, HEL was positively correlated with disease duration in RRMS (R = 0.25, *p* = 0.003) and negatively correlated with EDSS in PMS (R = −0.28, *p* = 0.02).

## 3. Discussion

In this study, we investigated circulatory molecular indicators of the main ferroptosis-related processes, including lipid peroxidation, antioxidant defense, and iron metabolism, with respect to the clinical course of MS. We found significantly decreased plasma levels of 4-HNE, total glutathione, and GSSG in patients with progressive MS compared to RRMS. The results indicate changes in redox processes associated with ferroptosis, steadily supporting the involvement of ferroptosis in key neuropathogenic mechanisms [45,46].

Among the most abundant and most studied lipid peroxidation products are MDA and 4-HNE [10,47]. More studies have examined and associated MDA with MS than 4-HNE. A meta-analysis of oxidative stress markers in MS, which included 31 studies and more than 2000 MS patients and healthy controls, demonstrated that patients with MS had significantly higher concentrations of both blood and CSF MDA compared with control subjects, supporting the role of oxidative lipid damage in neuropathogenic processes in MS [11]. Similarly, increased MDA measured in the CSF of newly diagnosed MS patients was correlated with brain atrophy [14]. We revealed no significant difference in plasma levels of MDA regarding the course of the disease. So far, significantly different MDA concentrations between RR and SP patients have been detected in CSF [48], while circulatory MDA was associated with neither cognitive function nor level of physical disability in MS patients [49]. Our findings propose that, in regard to MS course, disease-modifying therapy could significantly reduce the levels of plasma MDA in PMS patients in comparison with those who did not receive it. Similarly, in another study, serum MDA levels were higher in RRMS patients taking no disease-modifying therapy than in those taking interferon beta [12], all suggesting the protective effects of immunomodulation on lipid peroxidation-related detrimental outcomes in MS.

The elevated circulatory levels of 4-HNE have been associated with the progression of some neurological diseases [50,51]. To our knowledge, there is a lack of studies investigating circulatory 4-HNE in MS patients. Intriguingly, we detected significantly lower levels of plasma 4-HNE in PMS than in RRMS patients. Decreased 4-HNE has been proposed as a predictor of the progression of chronic diseases, including steroid-induced osteonecrosis [52] and hepatocellular carcinoma [53]. However, the findings of experimental models of MS, such as PMS-EAE, have shown an increase in 4-HNE levels in the CNS [15,16]. Immunohistochemical analysis of post-mortem cerebellar gray matter from subjects with MS, mostly progressive, indicated a link between 4-HNE and the demyelination process. However, the latter should be taken with caution, as only eight patients were included in the analysis [54]. In this study, we found that RR patients who self-reported fatigue had higher 4-HNE plasma concentrations than PMS patients with fatigue. Increased 4-HNE has been associated with fatigue in a model of chronic fatigue syndrome [55].

This is the first study to examine HEL, a more recently discovered, early-stage lipid peroxidation molecular indicator [17], in association with MS. There was no change in serum HEL concentrations between the two investigated subgroups of MS patients. Likewise, CSF and urinary HEL showed no significant associations with human acute encephalopathy/febrile seizures and severe motor and intellectual disabilities, respectively [56,57]. Interestingly, we found that both RR and progressive patients who were under therapy had approximately the same levels of HEL, while HEL was decreased in the progressive group among patients who did not receive therapy. Further analysis according to the type of disease-modifying treatment revealed that in RRMS subjects, HEL levels were significantly lower in those receiving interferon beta and glatiramer acetate compared to those receiving anti-CD20 antibody treatment. To our knowledge, this is the first study to observe the differential effects of various types of immunomodulatory therapy on circulating molecular parameters associated with lipid peroxidation, particularly within one course of MS. Hence, it would be worthwhile to evaluate this preliminary result further in a larger, well defined, and prospectively followed cohort of MS patients.

Out of three currently measured lipid peroxidation markers, 4-HNE levels were different according to disease course, with a significant decrease observed in PMS patients. Based on the previously mentioned studies, we expected to find higher 4-HNE levels in PMS than in RRMS patients. However, our results showed the opposite. One possibility for explaining the detected decrease in circulating 4-HNE would be an increase in cellular 4-HNE-protein adduct formation [58] due to excessive oxidative stress established in the progressive course of MS [59], along with assumed less efficient 4-HNE metabolism. Excessive formation of 4-HNE-protein adducts causes impaired function of many cellular proteins and is attributable to neurodegeneration [58,60]. This is a key feature of PMS and is in line with the fact that 4-HNE is the most toxic product of lipid peroxidation [61]. Given that under conditions of stress, 4-HNE is metabolized by aldehyde or alcohol dehydrogenase [62], these enzymes may become potential new therapeutic targets in MS, particularly with respect to disease course. On the other hand, the decrease in 4-HNE might reflect a potential secondary, compensatory response to counteract excessive oxidative stress that is expected in the progressive course of MS [59] since our recent study has shown upregulated expression of both pro- and antioxidant genes in SP compared to RR patients [63]. In support of this assumption, we observed a negative correlation of both 4-HNE and HEL with EDSS, exclusively in PMS patients. While 4-HNE was significantly changed, levels of the other investigated secondary lipid peroxidation product, MDA, were not different between the two patient groups, possibly pointing to differences that exist with regard to precursor PUFA as well as key enzymes involved in the formation and metabolism of MDA and 4-HNE [10]. We therefore propose that all these molecular components be further studied in the context of the MS disease course.

We demonstrated that GSSG and total glutathione were lower however, GSH and GPX4 levels remained unchanged in the plasma of progressive patients in comparison with RR. The latter may also reflect the unchanged efficacy of GSH and its related antioxidant system, including GPX4 and other enzymes, which participate in the removal of diverse lipid peroxidation products, along with those analyzed in this study [10]. A study of CSF and serum metabolome characterization in patients affected by RRMS and PPMS revealed that glutathione metabolites were among the most altered in serum between the two patient classes [64]. Cerebral GSH mapping showed a markedly lower GSH in PMS than RRMS [65]. In addition, SPMS patients with worsening clinical status, determined based on EDSS and senso-motor changes, had significantly greater declines in GSH concentrations than those with stable clinical status [66], indicating the prominent involvement of oxidative stress in the progressive stage of the disease. Our analysis of interactive effects demonstrated that an inverse relation between EDSS and GSH levels existed in RRMS. Furthermore, in RRMS patients, we observed a negative correlation between GPX4 levels and age, whereas GSSG was positively correlated with both age and BMI, pointing to an expected link between pro-oxidant status and increasing age/BMI [67,68]. Hence, based on the current findings, we propose that treatment with agents acting to increase GSH, such as fumarate [69], might be beneficial, particularly for RRMS patients. The previously mentioned studies that investigated GSH levels in the brain were performed on a limited number of participants, which should also be taken into consideration when interpreting results [65,66]. Altogether, further investigation to monitor changes simultaneously in the CNS and the periphery on a larger sample size is warranted to elucidate the impact of the glutathione antioxidant system on the pathogenesis and progression of MS.

Ferroptosis occurs when iron-related free radicals trigger lipid peroxidation under conditions of glutathione insufficiency and reduced lipid damage repair due to diminished GPX4 activity [70]. Therefore, in the context of ferroptosis, the absence of differences with respect to the majority of currently examined lipid peroxidation markers and glutathione antioxidant system components may assume no differences in iron metabolism indicators either. Indeed, we obtained no significant changes in serum levels of iron, transferrin, and ferritin between the two analyzed groups, RRMS and PMS patients. Several studies have shown no significant change, or even a decrease, in serum iron in MS patients compared to healthy controls [71,72]. However, iron deposition in the cerebral gray matter of MS patients was significantly higher than that of controls [72], and there was a significant relationship between iron concentrations in serum and subcortical deep gray matter in patients alone [31]. An early study revealed no changes in iron or transferrin levels in the CSF between MS and controls [73], while CSF/serum ferritin levels were significantly elevated in chronic progressive MS patients compared to controls and RR patients [43,73]. We found a significant negative correlation between serum ferritin and both EDSS and MSSS, whereby transferrin levels were positively correlated with MSSS. Since ferritin stores iron and transferrin participates in iron transport in and out of cells, correlations might reflect a mechanism to protect against free iron-induced oxidative inflammatory injury. Interestingly, we obtained the correlations in RR, and not progressive, MS patients, suggesting possible protective effects regarding the severity of the RRMS course. Transferrin was significantly decreased in the CSF of clinically isolated syndrome and early MS patients compared to controls, but with no significant differences observed in serum [36]. Higher CSF transferrin levels were correlated with lower physical disability scores [36], which is not in line with our findings. Furthermore, serum transferrin was lower and more frequently subnormal in PP patients in comparison with SP and RR patients [37]. We likewise found somewhat decreased transferrin in the overall group of progressive patients, corroborating the statistical trend. In contrast to the assumed protective effects of ferritin, which is the main free iron storage protein, serum ferritin, and lipid hydroperoxides showed an association with MS development [38], and MS patients with elevated ferritin had higher disease progression and advanced oxidation protein product levels and lower total radical-trapping antioxidant parameters than patients with lower ferritin [41]. Taken together, the current study and previous studies indicate that the action of molecular components involved in iron metabolism in MS differs in relation to disease course and stage as well as type of biological sample, while the rather heterogeneous study results denote that further investigation in this research field is warranted.

The present study is among the few that analyze multiple circulatory molecular indicators of the key ferroptosis-related processes in MS, with particular interest in finding the differences between the RR and the progressive course of the disease. The differential relations according to disease course were established for the administration of immunomodulatory therapy, the occurrence of fatigue, or level of disability regarding the plasma/serum concentrations of lipid peroxidation products (MDA, 4-HNE, and HEL) and glutathione. Still, circulatory levels of most of the analyzed components were not significantly different between the two study groups. These findings suggest that processes associated with ferroptosis may have a fairly strong and concomitant contribution to neuroinflammation and neurodegeneration, predominantly occurring in RRMS and PMS, respectively [4,28]. To our knowledge, this is the first study that collectively examined the main molecular indicators of all three key ferroptosis-related processes—lipid peroxidation, glutathione-mediated antioxidant defense, and iron metabolism—in patients with MS, which is the main strength of this research. Out of the target molecular indicators, HEL was investigated in MS for the first time, which enabled the monitoring of both the early and late stages of lipid peroxidation. Although a considerable number of patients were involved in the study, a constant limitation is the impossibility of the inclusion of equal numbers of patients with mild and severe progressive disease. Because of technical limitations, we could not perform the analysis of the glutathione system in a whole study group, which is another limitation regarding the number of patients. Due to a lack of detailed data regarding comorbidities, we were not able to investigate the possible links between molecular parameters and comorbidities in MS. This analysis would be beneficial since several studies have shown associations with depression and anxiety [74,75,76], which are major reported MS comorbidities [77]. Moreover, the existence of their precise causal influence on target molecular parameters has not been determined, thus requiring further research. We consider the lack of CSF investigation in this study group as a limitation that should be overcome in future studies. Most previous studies in MS have examined the currently analyzed molecular components either in the CNS or the periphery and within limited subject groups. Therefore, regarding the circulatory levels and relations of the target indicators of ferroptosis, the differences between relapsing–remitting and progressive MS patients described here support further investigation of the impact of ferroptosis-related processes and their molecular markers, both in the CNS and periphery, on pathogenesis and progression of MS as well as on defining potential therapeutics effective in improving the redox state in patients with MS.

## 4. Materials and Methods

### 4.1. Subjects

The overall study group comprised 222 unrelated patients from Serbia: 153 with RRMS, 54 with SPMS, and 15 with PPMS. Patients were recruited from the Clinic for Neurology of Military Medical Academy (MMA), Belgrade, Serbia, and the Neurology Clinic of University Clinical Center Nis, Nis, Serbia, during their regular visits to the clinics in the period 2022–2023. MS was diagnosed to fulfill revised McDonald criteria [78], and the course of disease was defined according to a clinical method [79,80,81,82]. A detailed questionnaire was filled out for each patient, based on clinical records and an interview with a neurologist, to provide accurate data on anthropometric and clinical parameters, which were determined at the time of peripheral blood sample collection. The anthropometric parameters included age, sex, body mass index (BMI), and smoking status. Clinical parameters included disease onset age, disease duration, EDSS, MSSS, the total number of registered relapses, and previous and ongoing therapy. Fatigue was taken into account as a self-reported, subjective parameter. The EDSS score, representing a measure of neurological disability based on clinical assessments, was used for the evaluation of the clinical severity of the disease [83]. The progression of disability was determined by MSSS, which corrects the EDSS for disease duration [84]. The main inclusion criteria were the following: diagnosed MS, disease duration of at least one year, and age between 18 and 65 years. Exclusion criteria were the following: relapse during the period of at least 30 days prior to the study enrollment and consequently, a recent corticosteroid treatment, clinically isolated or radiologically isolated syndrome, comorbidities for neurological diseases other than MS and autoimmune diseases, diagnosed malignancies, pregnancy and inability to sign informed consent. Patients having acute infections were also not enrolled in the study. All patients had similar dietary habits that included meat, vegetables, and fruit intake. Besides vitamin D, there were no continuous supplementations at least three months before blood collection that could influence the measured parameters.

Out of 139 RR patients under therapy, the majority had been receiving a low-risk, moderate-efficacy disease-modifying therapy, including interferon beta and glatiramer acetate medicines, for at least 24 months (except newly diagnosed patients who represented less than 5% of the total number of patients). In those with a progression of the disease, initial therapy was replaced with S1PR modulator or CD20-directed monoclonal antibody. Among the progressive patients (SP and PP), 50 were receiving the moderate-efficacy immunomodulatory treatment during the period of at least 12 months, followed by the more efficacious therapeutics after disease breakthroughs or stopping the treatment on several accounts. In all progressive patients, the disease has progressed despite the therapy. Fourteen (14) RRMS and 19 progressive patients were treatment-naive.

The MMA Ethics Committee approved the study (Decision No. 6/2020), and each participant gave their written informed consent for participation in the study.

### 4.2. Quantification of MDA, 4-HNE, GPX4 and Glutathione in Plasma

Peripheral blood samples, collected with EDTA, were centrifuged at 1000× *g* for 15 min at +4 °C within 30 min of collection. The supernatant plasma was collected and stored at −20 °C prior to use.

FineTest^®^ kits for enzyme-linked immunosorbent assay (ELISA): Human MDA (Malondialdehyde) ELISA Kit, Human 4-HNE (4-Hydroxynonenal) ELISA Kit and Human GPX4 (Phospholipid hydroperoxide glutathione peroxidase) ELISA Kit (Wuhan Fine Biotech Co., Ltd., Wuhan, China), were used for quantification of MDA, 4-HNE, and GPX4, respectively, in plasma samples of all 222 patients. The optical density (OD) of samples was measured at 450 nm by using a PKL PPC 142 ELISA reader (PKL^®^ POKLER ITALIA, PARAMEDICAL srl, Salerno, Italy), and the OD values were used for determination of MDA (ng/mL), 4-HNE (pg/mL) and GPX4 (pg/mL) concentration from a four parameter logistic (4PL) curve [85].

Quantification of oxidized (GSSG), reduced (GSH) and total (GSH + GSSG) glutathione was carried out in plasma samples of 100 patients, 63 with RR (34 women and 29 men) and 37 with progressive (25 women and 12 men) course of MS, representing a subgroup of the initial group of 222 patients, by using a Sigma-Aldrich^®^ Quantification kit for oxidized and reduced glutathione (Sigma-Aldrich, Merck KGaA, Darmstadt, Germany). The GSSG and total glutathione concentrations (μmol/L) in samples were determined from corresponding linear regression curves [86] constructed by using the OD values of standards and samples, derived from a colorimetric reaction and measured at 405 nm (PKL PPC 142 ELISA reader, PKL^®^ POKLER ITALIA, PARAMEDICAL srl, Salerno, Italy). The quantity of GSH (μmol/L) was determined by subtracting the amount of GSSG from the total amount of glutathione, according to the manufacturer’s instructions.

### 4.3. Quantification of HEL, Iron, Transferrin, and Ferritin in Serum

After the collection of peripheral blood, samples were allowed to be clotted by leaving the blood undisturbed at room temperature for 2 h. The clot was removed by centrifugation at 1000× *g* for 20 min at +4 °C, and the supernatant serum was collected and stored at −20 °C prior to use. Serum HEL was measured using the JaICA Hexanoyl-Lys adduct (HEL) ELISA kit (Japan Institute for the Control of Aging (JaICA), Nikken SEIL Co., Ltd., Haruoka, Fukuroi, Shizuoka, Japan). The enzyme pretreatment of 222 serum samples and the following assay procedure were performed according to the manufacturer’s instructions. The OD values of samples were measured at 450 nm (PKL PPC 142 ELISA reader, PKL^®^ POKLER ITALIA, PARAMEDICAL srl, Salerno, Italy) and used for the determination of the HEL concentration (nmol/L) from a four-parameter logistic (4PL) curve [85].

The iron concentrations (µmol/L) in 222 serum samples were determined using spectrophotometry, and transferrin (g/L) was measured through immunoturbidimetry using a URIT-8210 Automatic clinical chemistry analyzer (URIT Medical Electronic Co., Ltd., Shenzhen, China). The ferritin serum concentration (ng/mL) was determined through immunoturbidimetry using an AutoLumo A1000 Chemiluminescence Immunoassay System (Autobio Diagnostics Co., Ltd., Zhengzhou, China).

### 4.4. Statistical Analysis

The statistical analysis of all the data was performed using the Statistica 8.0 software package (StatSoft, Inc., Tulsa, OK, USA). Differences in the distributions of categorical variables, including sex and status of smoking, fatigue, and therapy, between the groups of patients with RR and progressive (SP + PP) MS were assessed using the Fisher exact test. The normality of the distribution of continuous variables, which encompassed the anthropometric, clinical, and molecular parameters (indicators of ferroptosis-related processes), was tested with the Kolmogorov–Smirnov test with Lilliefors correction and the Shapiro–Wilk test. Comparisons of continuous variable values between the patient groups were performed using a *t*-test or Mann–Whitney U test, depending on whether the values had a normal distribution or not. For uniform presentation, the values of all the continuous variables are provided as the median (minimum–maximum). Factorial ANOVA was performed for the estimation of the interactive effects of disease course (RR/progressive) and other relevant categorical variables (sex, status of therapy, fatigue, and smoking) on the levels of assessed molecular parameters in MS patients. Disease course and continuous anthropometric/clinical parameters interaction effects on molecular parameters in MS patients were assessed through ANCOVA. The correlations of anthropometric and clinical parameters with molecular parameters in each patient group, with RR and progressive MS, were estimated with the Spearman rank order correlation test. In all tests, *p* < 0.05 values were considered significant. For the graphical presentation of results, Prism v6.01 software (GraphPad Software, Inc., Boston, MA, USA) was employed.

## Figures and Tables

**Table 1:** Anthropometric and clinical parameters of MS patients with regard to disease course.

Anthropometric and Clinical Parameters	RRMS n = 153	PMS n = 69	*p*-Value
Sex (women/men, n)	79/74	42/27	0.25 ^$^
Age (years)	43.0 ± 9.8	49.1 ± 7.9	2 × 10^−5 #^
Body mass index (BMI, kg/m^2^)	24.40 ± 3.92	24.19 ± 4.12	0.67 ^&^
Smoking status (yes/no/no data, n)	51/97/5 ^†^	24/42/3 ^†^	0.88 ^$^
Disease onset age (years)	32.6 ± 9.2	34.2 ± 10.2	0.43 ^&^
Disease duration (years)	10.4 ± 5.9	14.8 ± 8.2	2 × 10^−4 &^
EDSS	1.8 ± 1.1	5.6 ± 1.2	<10^−6 &^
MSSS	2.32 ± 1.79	6.22 ± 1.83	<10^−6 &^
Total number of relapses	2.00 (0.00–17.00)	6.00 (0.00–18.00)	<10^−6 &^
Fatigue (yes/no/no data, n)	75/61/17 ^†^	54/10/5 ^†^	6 × 10^−5 $^
Therapy (yes/no, n)	139/14	50/19	3 × 10^−4 $^

RRMS—relapsing–remitting multiple sclerosis; PMS—progressive multiple sclerosis; n—number of patients; EDSS—Expanded Disability Status Scale; MSSS—Multiple Sclerosis Severity Score; ^$^ Fisher exact test; ^#^ *t*-test; ^&^ Mann–Whitney U test; values of continuous parameters are presented as mean ± standard deviation except for the total number of relapses (presented as median, minimum and maximum); *p*-value: comparison PMS vs. RRMS; *p*-values < 0.05 were considered statistically significant; ^†^ patients with no available data were not included in the analysis.

**Table 2:** Molecular parameters in patients with MS according to disease course.

Molecular Parameters	RRMS n = 153	PMS n = 69	*p*-Value
Malondialdehyde (MDA, ng/mL)	98.94 ± 40.71	103.77 ± 67.50	0.89
4-Hydroxynonenal (4-HNE, pg/mL)	1848.24 ± 1115.45	1712.27 ± 1289.24	0.03
Hexanoyl-lys adduct (HEL, nmol/L)	12.68 ± 3.93	13.17 ± 3.77	0.39
Glutathione peroxidase 4 (GPX4, pg/mL)	2503.18 ± 1606.99	2442.53 ± 1349.04	0.83
* Total glutathione (GSH + GSSG, μmol/L)	44.46 ± 6.48	42.03 ± 11.26	0.006
* Reduced glutathione (GSH, μmol/L)	15.77 ± 7.30	16.35 ± 8.13	0.91
* Oxidized glutathione (GSSG, μmol/L)	14.35 ± 2.47	12.84 ± 3.41	0.003
* Reduced/oxidized glutathione (GSH/GSSG)	1.18 ± 0.69	1.34 ± 0.69	0.17
Iron (Fe, µmol/L)	14.63 ± 5.34	14.60 ± 4.63	0.85
Transferrin (Tf, g/L)	2.53 ± 0.36	2.42 ± 0.43	0.07
Ferritin (Ft, ng/mL)	65.71 ± 61.58	75.39 ± 116.71	0.56

RRMS—relapsing–remitting multiple sclerosis; PMS—progressive multiple sclerosis; n—number of patients; values of continuous parameters are presented as means ± standard deviations; *p*-value (Mann–Whitney U test): comparison PMS vs. RRMS; *p*-values < 0.05 were considered statistically significant; * analysis performed in a subgroup of 100 patients, 63 RRMS and 37 PMS.

**Table 3:** Interactive effects of disease course (RRMS/PMS) and sex, therapy, fatigue or smoking status on levels of molecular parameters in MS patients.

Molecular Parameters	MS Course × Sex F *p*-Value	MS Course × Th F *p*-Value	MS Course × Fatigue F *p*-Value	MS Course × Smoking F *p*-Value
Malondialdehyde (MDA, ng/mL)	0.13 0.72	7.13 0.009	0.10 0.75	0.00 0.99
4-Hydroxynonenal (4-HNE, pg/mL)	0.92 0.34	0.21 0.65	6.19 0.01	2.02 0.16
Hexanoyl-lys adduct (HEL, nmol/L)	0.07 0.78	5.77 0.02	0.50 0.48	0.66 0.42
Glutathione peroxidase 4 (GPX4, pg/mL)	0.63 0.43	0.48 0.49	0.57 0.45	0.52 0.47
Total glutathione (GSH + GSSG, μmol/L)	0.51 0.47	0.68 0.41	0.10 0.76	0.51 0.47
Reduced glutathione (GSH, μmol/L)	1.35 0.25	0.10 0.75	2.99 0.09	0.47 0.49
Oxidized glutathione (GSSG, μmol/L)	0.09 0.76	0.08 0.78	2.51 0.12	2.63 0.11
Reduced/oxidized glutathione (GSH/GSSG)	1.16 0.28	0.03 0.87	4.27 0.04	1.40 0.24
Iron (Fe, µmol/L)	0.84 0.36	0.65 0.42	2.78 0.10	0.33 0.57
Transferrin (Tf, g/L)	0.41 0.52	0.004 0.95	0.007 0.94	1.33 0.25
Ferritin (Ft, ng/mL)	0.18 0.67	2.15 0.15	0.46 0.50	1.91 0.17

Factorial ANOVA was performed for estimation of the interactive effects; values of all molecular parameters were log (2) transformed, except those for iron; n—number of patients; F—the ratio of the between-group variance to the within-group variance; *p*-values < 0.05 were considered statistically significant. Th—therapy.

**Table 4:** Analysis of the relationship between disease course (RRMS/PMS), anthropometric or clinical parameters and levels of molecular parameters in MS patients.

Molecular Parameters	MS Course F *p*-Value	Age (y) F *p*-Value	BMI (kg/m^2^) F *p*-Value	Disease Durat. (y) F *p*-Value	EDSS F *p*-Value	MSSS F *p*-Value
Malondialdehyde (MDA, ng/mL)	0.60 0.44	0.03 0.87	1.65 0.20	2.33 0.13	2.93 0.09	3.11 0.08
4-Hydroxynonenal (4-HNE, pg/mL)	1.98 0.16	0.01 0.90	0.51 0.48	0.0002 0.99	0.11 0.74	0.15 0.70
Hexanoyl-lys adduct (HEL, nmol/L)	0.26 0.61	0.04 0.84	0.11 0.74	0.18 0.67	0.02 0.88	0.00 1.00
Glutathione peroxidase 4 (GPX4, pg/mL)	0.07 0.79	0.42 0.52	0.02 0.90	0.003 0.96	0.08 0.77	0.09 0.77
Total glutathione (GSH + GSSG, μmol/L)	0.78 0.38	0.007 0.93	0.08 0.78	0.45 0.51	2.11 0.15	1.07 0.30
Reduced glutathione (GSH, μmol/L)	4.94 0.03	0.03 0.87	1.08 0.30	3.02 0.09	4.29 0.04	2.29 0.13
Oxidized glutathione (GSSG, μmol/L)	1.11 0.29	0.46 0.50	1.30 0.26	0.88 0.35	0.0005 0.98	0.003 0.96
Reduced/oxidized Glutathione (GSH/GSSG)	5.37 0.02	0.18 0.67	1.82 0.18	3.46 0.07	3.11 0.08	1.71 0.19
Iron (Fe, µmol/L)	1.00 0.32	4.00 0.05	0.80 0.37	0.24 0.63	0.59 0.44	0.58 0.45
Transferrin (Tf, g/L)	0.00 1.00	0.50 0.48	1.11 0.29	0.25 0.62	0.44 0.51	0.49 0.48
Ferritin (Ft, ng/mL)	0.004 0.95	1.84 0.18	1.17 0.28	0.24 0.62	0.31 0.58	1.01 0.32

ANCOVA was performed to assess the interaction effects of disease course and anthropometric and clinical parameters on molecular parameters; values of continuous parameters were log (2) transformed, except age and iron; BMI—body mass index; EDSS—Expanded Disability Status Scale; MSSS—Multiple Sclerosis Severity Score; F—the ratio of the between-group variance to the within-group variance; *p*-values < 0.05 were considered statistically significant.

**Table 5:** Correlations of anthropometric and clinical parameters with molecular parameters in RRMS (**a**) and PMS (**b**) patients.

**(a)**					
**Molecular Parameters**	**Age (Years)** **R** ***p*-Value**	**BMI** **(kg/m^2^)** **R** ***p*-Value**	**Disease** **Duration (y)** **R** ***p*-Value**	**EDSS** **R** ***p*-Value**	**MSSS** **R** ***p*-Value**
Malondialdehyde (MDA, ng/mL)	−0.04 0.65	−0.11 0.18	0.06 0.49	0.02 0.76	−0.04 0.67
4-Hydroxynonenal (4-HNE, pg/mL)	0.05 0.58	−0.11 0.20	−0.09 0.30	−0.08 0.37	−0.02 0.78
Hexanoyl-lys adduct (HEL, nmol/L)	−0.005 0.95	−0.16 0.06	0.25 0.003	0.12 0.13	0.01 0.86
Glutathione peroxidase 4 (GPX4, pg/mL)	−0.24 0.004	0.05 0.53	−0.10 0.25	−0.02 0.80	0.03 0.68
Total glutathione (GSH + GSSG, μmol/L)	0.006 0.96	−0.03 0.84	−0.05 0.68	−0.22 0.08	−0.13 0.30
Reduced glutathione (GSH, μmol/L)	−0.15 0.24	−0.19 0.16	0.07 0.58	−0.21 0.10	−0.17 0.17
Oxidized glutathione (GSSG, μmol/L)	0.27 0.03	0.29 0.02	−0.19 0.15	0.08 0.51	0.17 0.18
Reduced/oxidized glutathione (GSH/GSSG)	−0.18 0.16	−0.22 0.09	0.12 0.37	−0.22 0.08	−0.20 0.11
Iron (Fe, µmol/L)	0.07 0.38	0.05 0.54	0.14 0.08	−0.10 0.21	−0.15 0.07
Transferrin (Tf, g/L)	−0.06 0.43	−0.05 0.52	−0.26 0.002	0.11 0.19	0.20 0.01
Ferritin (Ft, ng/mL)	0.04 0.59	0.21 0.01	0.13 0.12	−0.19 0.02	−0.19 0.02
**(b)**					
**Molecular ** **Parameters**	**Age (Years)** **R** ***p*-Value**	**BMI** **(kg/m^2^)** **R** ***p*-Value**	**Disease** **Duration (y)** **R** ***p*-Value**	**EDSS** **R** ***p*-Value**	**MSSS** **R** ***p*-Value**
Malondialdehyde (MDA, ng/mL)	−0.02 0.90	0.07 0.59	0.009 0.94	−0.03 0.80	−0.0004 1.00
4-Hydroxynonenal (4-HNE, pg/mL)	−0.25 0.05	0.14 0.29	−0.12 0.35	−0.27 0.03	−0.06 0.62
Hexanoyl-lys adduct (HEL, nmol/L)	0.01 0.92	−0.15 0.23	−0.15 0.22	−0.28 0.02	−0.01 0.94
Glutathione peroxidase 4 (GPX4, pg/mL)	−0.13 0.31	0.19 0.14	0.12 0.35	0.23 0.06	0.23 0.06
Total glutathione (GSH + GSSG, μmol/L)	−0.09 0.61	−0.02 0.93	−0.12 0.49	−0.20 0.25	0.04 0.82
Reduced glutathione (GSH, μmol/L)	0.23 0.17	0.10 0.55	0.25 0.13	−0.01 0.95	−0.005 0.98
Oxidized glutathione (GSSG, μmol/L)	−0.28 0.09	0.0005 1.00	−0.23 0.17	−0.32 0.05	−0.11 0.50
Reduced/oxidized glutathione (GSH/GSSG)	0.29 0.08	0.05 0.78	0.30 0.07	0.18 0.29	0.07 0.70
Iron (Fe, µmol/L)	0.30 0.01	0.11 0.38	−0.08 0.54	0.04 0.76	0.04 0.77
Transferrin (Tf, g/L)	−0.24 0.06	−0.13 0.31	−0.07 0.58	−0.11 0.39	0.03 0.83
Ferritin (Ft, ng/mL)	0.17 0.17	0.27 0.03	−0.09 0.48	0.06 0.61	0.11 0.36

BMI—body mass index; y—years; EDSS—Expanded Disability Status Scale; MSSS—Multiple Sclerosis Severity Score; R—Spearman’s rank correlation coefficient; *p*-values < 0.05 were considered statistically significant.

## Data Availability

The original contributions presented in the study are included in the article/Supplementary Materials; further inquiries can be directed to the corresponding author.

## Supplementary Materials

The following supporting information can be downloaded at: https://www.mdpi.com/article/10.3390/ijms252011024/s1.
