# Supplementary material for: Circulatory Indicators of Lipid Peroxidation, the Driver of Ferroptosis, Reflect Differences between Relapsing–Remitting and Progressive Multiple Sclerosis
Source: Int J Mol Sci. 2024 Oct 14;25(20):11024. doi: 10.3390/ijms252011024 (PMC11507982; doi:10.3390/ijms252011024)
Supplement: Supplementary file 1 [file ijms-25-11024-s001.zip › ijms-3190575-supplementary.pdf]

a)

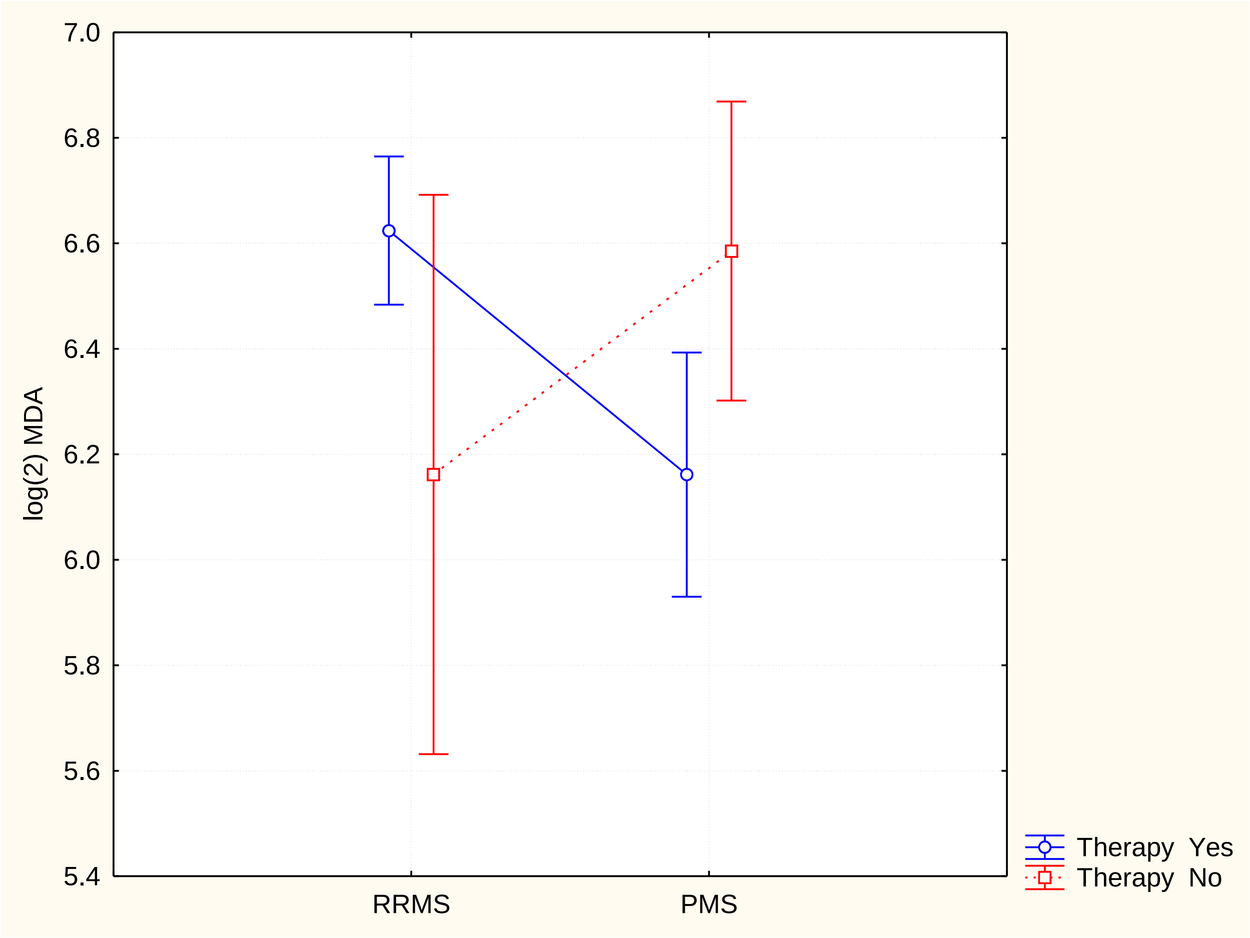

7.0
6.8
6.6
6.4
6.2
6.0
5.8
5.6
5.4
log(2) MDA
RRMS
PMS
Therapy Yes
Therapy No

b)

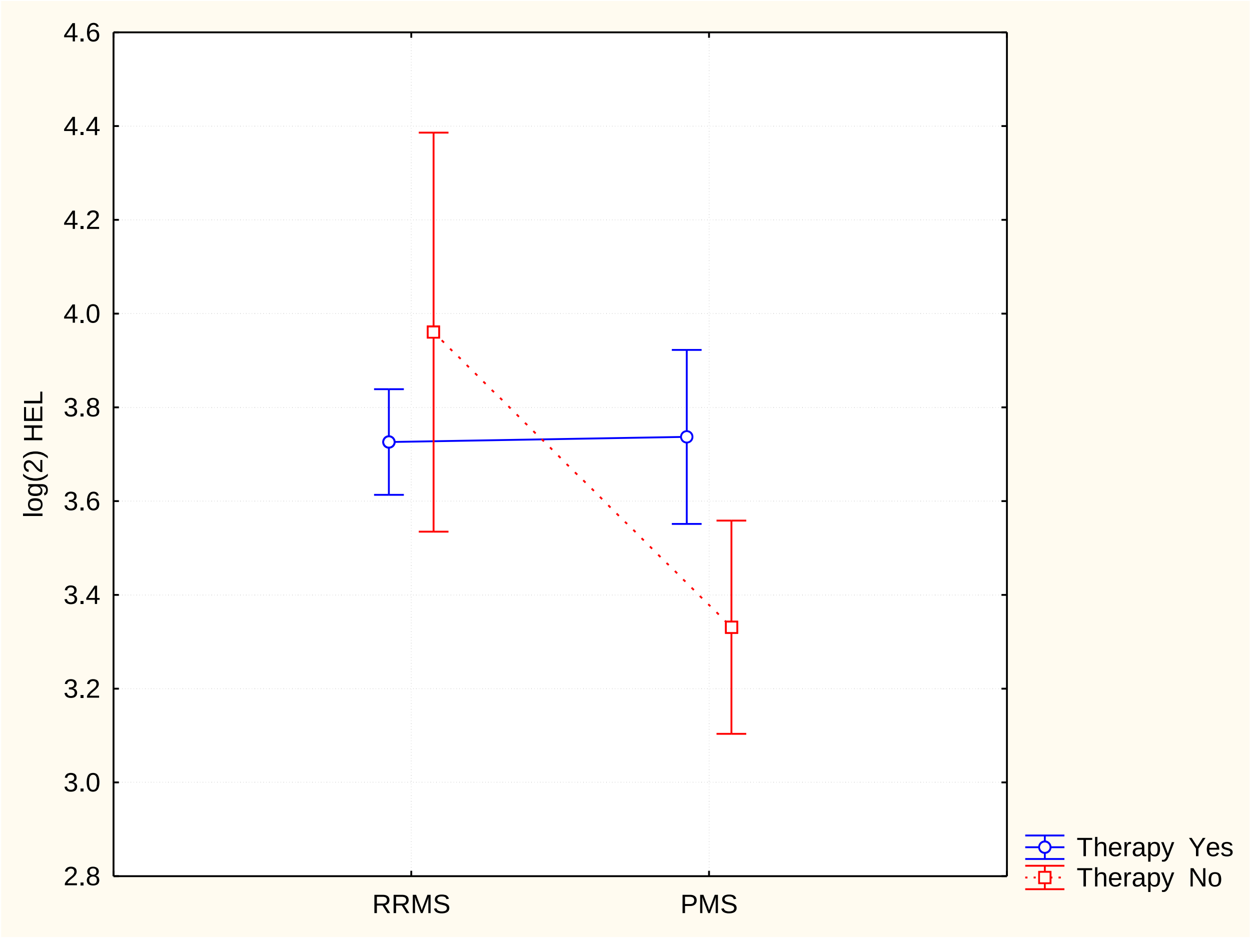

4.6
4.4
4.2
4.0
3.8
3.6
3.4
3.2
3.0
2.8
log(2) HEL
RRMS
PMS
Therapy Yes
Therapy No

c)

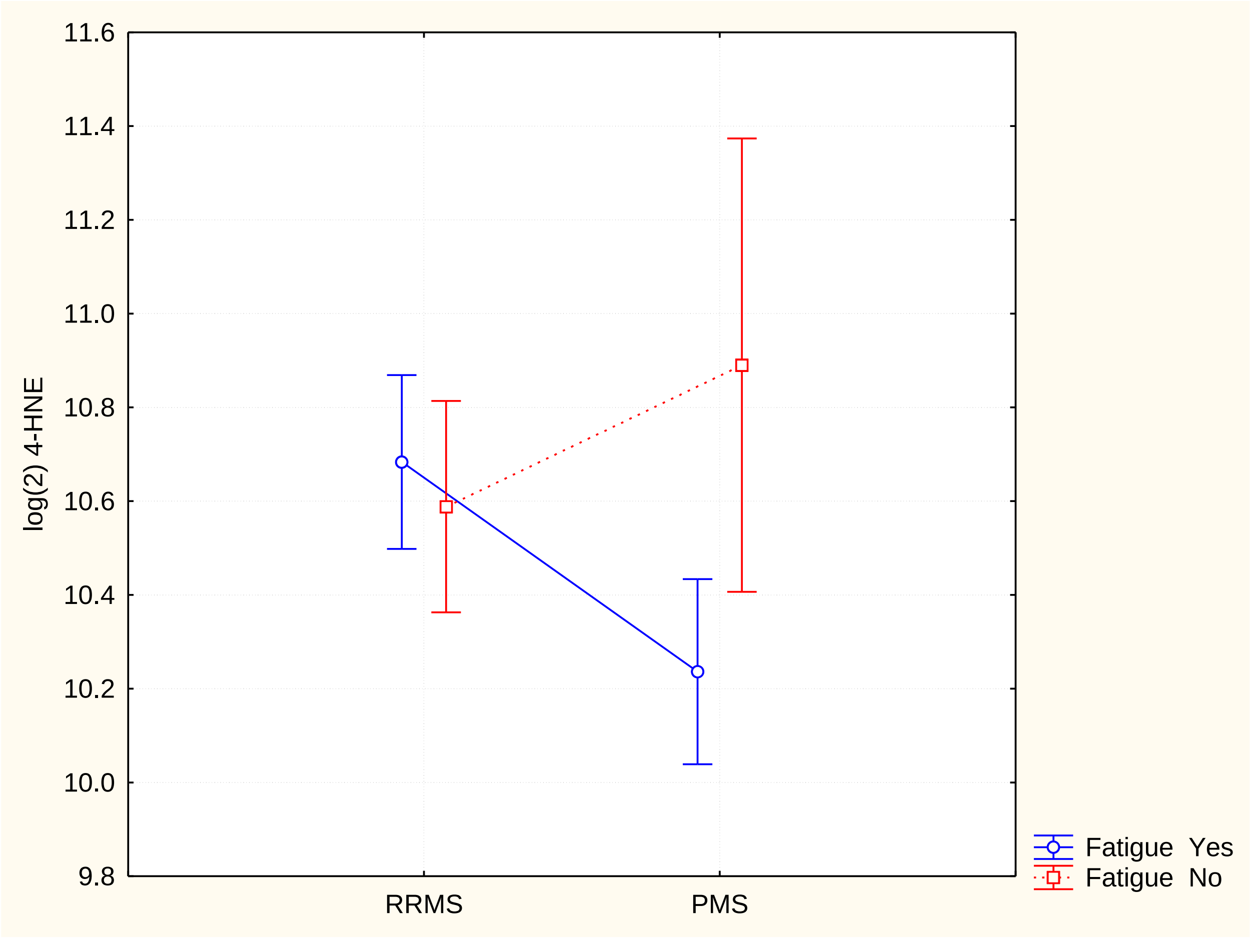

11.6
11.4
11.2
11.0
10.8
10.6
10.4
10.2
10.0
9.8
log(2) 4-HNE
RRMS
PMS
Fatigue Yes
Fatigue No

Figure S1. Factorial ANOVA for the interactive effects of:

a) MS course (RRMS/PMS) and current therapy status (receiving/not receiving) on MDA levels ($F = 7.13$, $p = 0.009$);
b) MS course and current therapy status on HEL levels ($F = 5.77$, $p = 0.02$);
c) MS course and self-reported fatigue (yes/no) on 4-HNE levels ($F = 6.19$, $p = 0.01$).

F–ratio of the between-group variance to the within-group variance; p-value < 0.05 was considered statistically significant.
Graph: LS means; vertical bars denote 0,95 confidence intervals; RRMS–relapsing-remitting multiple sclerosis; PMS–progressive multiple sclerosis.
